# Agreement between rhinomanometry and computed tomography-based computational fluid dynamics

**DOI:** 10.1007/s11548-021-02332-1

**Published:** 2021-03-07

**Authors:** Manuel Berger, Aris I. Giotakis, Martin Pillei, Andreas Mehrle, Michael Kraxner, Florian Kral, Wolfgang Recheis, Herbert Riechelmann, Wolfgang Freysinger

**Affiliations:** 1 Department of Environmental, Process and Energy Engineering, MCI, The Entrepreneurial School, Innsbruck, Austria; 2 Department of Otorhinolaryngology, Medical University of Innsbruck, Innsbruck, Austria; 3 Department of Fluid Mechanics, Friedrich-Alexander-University Erlangen-Nuremberg, Erlangen, Germany; 4 Department of Mechatronics, MCI, The Entrepreneurial School, Innsbruck, Austria; 5 University Hospital of Radiology, Medical University of Innsbruck, Innsbruck, Austria

**Keywords:** Computational fluid dynamics, Nasal obstruction, Rhinomanometry, Simulation, Agreement analysis, Method comparison

## Abstract

**Purpose:**

Active anterior rhinomanometry (AAR) and computed tomography (CT) are standardized methods for the evaluation of nasal obstruction. Recent attempts to correlate AAR with CT-based computational fluid dynamics (CFD) have been controversial. We aimed to investigate this correlation and agreement based on an in-house developed procedure.

**Methods:**

In a pilot study, we retrospectively examined five subjects scheduled for septoplasty, along with preoperative digital volume tomography and AAR. The simulation was performed with Sailfish CFD, a lattice Boltzmann code. We examined the correlation and agreement of pressure derived from AAR (RhinoPress) and simulation (SimPress) and these of resistance during inspiration by 150 Pa pressure drop derived from AAR (RhinoRes150) and simulation (SimRes150). For investigation of correlation between pressures and between resistances, a univariate analysis of variance and a Pearson’s correlation were performed, respectively. For investigation of agreement, the Bland–Altman method was used.

**Results:**

The correlation coefficient between RhinoPress and SimPress was *r* = 0.93 (*p* < 0.001). RhinoPress was similar to SimPress in the less obstructed nasal side and two times greater than SimPress in the more obstructed nasal side. A moderate correlation was found between RhinoRes150 and SimRes150 (*r* = 0.65; *p* = 0.041).

**Conclusion:**

The simulation of rhinomanometry pressure by CT-based CFD seems more feasible with the lattice Boltzmann code in the less obstructed nasal side. In the more obstructed nasal side, error rates of up to 100% were encountered. Our results imply that the pressure and resistance derived from CT-based CFD and AAR were similar, yet not same.

**Supplementary Information:**

The online version contains supplementary material available at 10.1007/s11548-021-02332-1.

## Introduction

Active anterior rhinomanometry (AAR) is an international standardized method for the evaluation of nasal obstruction [1, 2]. AAR measures nasal pressure and airflow during inspiration and expiration. Specifically, it measures pressure differences between the nasal entrance (ambient pressure) and the nasopharynx. AAR can be influenced by alternating congestion and decongestion of the nasal mucosa, a physiologic process that is termed “nasal cycle” [3, 4]. The nasal cycle results into different mucosal congestion status between the left and right nasal side [5]. Another important tool for evaluation of nasal obstruction is computed tomography (CT).

Recently, the importance of computational fluid dynamics (CFD) in rhinology has been highlighted [6]. CFD uses numerical methods to analyze and solve problems that involve fluid flows, e.g., aerodynamics. In their recent review, Leite and coauthors concluded that CFD may become a viable diagnostic tool in the future for studying nasal physiology [7]. Recently, Radulesco and coauthors searched for data to compare nasal obstruction with CFD variables. Among CFD-calculated resistances, airflow, heat flux, wall shear stress, total pressure, velocities and streamlines, the authors reported the heat flux as the most correlated CFD variable with subjective nasal obstruction. Total pressure and velocities were also useful [8].

In an attempt to investigate the correlation between AAR and CT-based CFD, Kaneda and coauthors compared the nasal patency computed by solving the Navier–Stokes equations with the AAR-measured nasal patency [9]. Despite the unsatisfactory correlation, the authors reported similar qualitative tendencies between both methods. Cherobin and coauthors investigated the correlation and agreement between the resistances derived from AAR and CFD [10]. The authors reported a weak correlation between the values of nasal resistance derived from AAR and CFD (*r* = 0.41, *p* = 0.003).

In our study, we investigated the correlations and agreements between the results from AAR and an in-house developed CT-based CFD approach for multiple subjects. Our CFD simulations of nasal airflow were previously validated with laser Doppler anemometry (LDA) [11]. LDA is a fluid flow measurement technique that captures the velocity of particles in the flow field. LDA is noninvasive and does not influence the flow field. Since fluid flow phenomena are difficult to simulate accurately (turbulence, separation), LDA was used to determine the accuracy of the simulated nasal airflow. LDA requires optical accessibility to the flow field. Therefore, in vivo measurements were not possible. A simplified 3D printed model (Dremel 3D20, Dremel, Racine, USA), based on a CT dataset of a septal deviation CT dataset, was investigated with LDA. Optical accessibility was guaranteed by acrylic glass elements. The measurement results were then compared to those of the LB simulations. On a line introduced near the nasal valve for evaluation, the maximum velocity difference between LDA and the LB simulation was below 15%, which was considered acceptable.

## Materials and methods

### Study design/sample

To address the research goals, we designed and implemented a pilot study. The study population was composed of adult patients presenting to the Department of Otorhinolaryngology for evaluation and management of nasal obstruction between 01/01/2019 and 31/12/2019. To be included in the study sample, patients had to be scheduled for septoplasty along with preoperative digital volume tomography (DVT) and preoperative AAR. Patients were excluded if sinus opacification or tumors were present. For this pilot study, we retrospectively selected five patients using our operation management software (MyMedis; Getinge, Rastatt, Germany). Starting from the last subject operated upon the study period, we sought applicable subjects going backwards in time and stopped after selecting five subjects. Septal deviation was documented after a consultation of the surgery reports.

All procedures performed in studies involving human participants were in accordance with the ethical standards of the institutional and/or national research committee and with the 1964 Helsinki Declaration and its later amendments or comparable ethical standards. This research study was conducted retrospectively from data obtained for clinical purposes. Informed consent was available for each patient as a part of a broad institutional-patient consent that was signed before treatment.

### Imaging parameters and visualization software

The DVT protocol (Imaging Sciences DVT; KaVo, Biberach/Riss, Germany) included a slice thickness of 0.3 mm, voxel size 0.3 × 0.3 × 0.3 mm^3^, and a matrix with 536 × 536 pixels. Syngo-share-view (Siemens Healthcare Diagnostics GmbH; Vienna, Austria) was used to visualize and extract the DICOM (digital imaging and communication in medicine)-format data sets.

### Active anterior rhinomanometry

The Otopront Rhino-Sys system (Otopront; Hohenstein, Germany) was used. Prior to the examination, each subject waited 15 min to become acclimatized to the indoor climate [12]. On average, three breathing cycles were acquired. Inspiratory and expiratory airflow (ml/s), flow increase (%) and inspiratory and expiratory resistance (sPa/ml) were automatically displayed, for the left nasal side, the right nasal side and bilaterally, before and 10 min after decongestion, with three puffs of nasal xylometazoline spray 0.05% per side. No subject used nasal xylometazoline spray on the examination day prior to AAR.

### Digitization of active anterior rhinomanometry

The AAR results were saved in pdf file format and digitized with WebPlotDigitizer 4.2 software (Automeris; San Francisco, California, USA). Pixel-based image data were converted to numerical *x*–*y* coordinates. The raw data were thresholded with RGB color for congested right (255, 223, 204) ± 10, congested left (103, 148, 198) ± 10, decongested right (232, 138, 135) ± 10 and decongested left (193, 218, 242) ± 10 to separate the AAR curves. A discretization window of 5 × 5 pixels was chosen; the picture resolution was 847 × 757 pixels. More than 1500 *x*–*y* points per AAR curve were saved in csv file format.

### Air space extraction

**Fig. 1:**
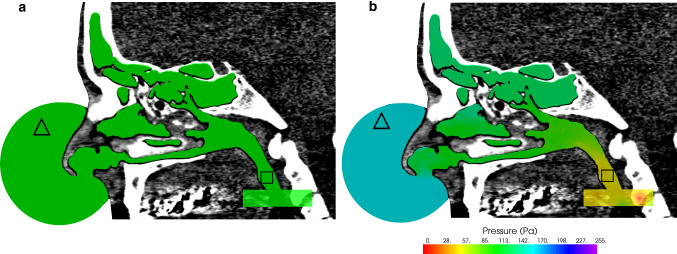
**a** Segmentation of the right nasal airspace in one subject. **Δ** sphere positioned at the nasal tip. □ cuboid in the oropharynx. **b** Pressure simulation outcome of the right nasal airspace in one subject. The color change (pressure gradient) refers to the pressure drop that indicates an airflow constriction. Cyan = ambient pressure level, green = pressure level within the nasal airway passage, yellow = pressure level at the oropharynx. **Δ** p1, □ p2

A fully automated Python script was programmed (packages: dicom, scipy.ndimage) for air-segmentation of the CT datasets. The nasal airspace was extracted as the basis for the CFD simulations. Thresholding to − 460 Hounsfield units (HU) [13] converted the integer-based CT dataset to binary: air voxels in the CT dataset were set to “1”, and all others were set to “0”. The nasal tip was automatically detected with the python script, defining the midpoint of a sphere with a diameter of 70 mm (Fig. 1a). The inlet boundary condition for the CFD simulation was set on the surface of this sphere. A cuboid (60 × 40 × 30 mm^3^) was positioned automatically onto the oropharynx to determine the AAR inspiration/expiration flow rate $\dot{V}$ for the CFD simulation (Fig. 1a). A region-growing algorithm extracted the nasal airspace by removing all other air voxels (Fig. 1a). We performed one single manual correction in order to reflect the AAR conditions. Here, the right nostril was blocked manually when the flow through the left nostril was investigated and vice versa.

### Simulation parameters

Sailfish CFD [14], a lattice Boltzmann (LB) code running on an Nvidia GeForce RTX 2080 Ti graphic processing unit (GPU), was used for simulation of the nasal airflow [11]. One nasal airflow simulation on the GPU required less than 5 min of computational time. To simulate the AAR curve, the flow rate was varied from 0 to 600 ml/s in steps of 50 ml/s for inspiration and expiration for each nostril. At the cuboid outlet area, a Dirichlet velocity boundary condition was applied to set the airflow. The fluid flow boundary condition at the sphere surface was set to ambient pressure. Nasal airflows tend to be turbulent at flow rates > 290 ml/s [15]. Therefore, the large eddy simulation with the Smagorinsky subgrid model (constant *c*_s_ = 0.14) [14] was used to model spatially/temporally unresolved eddies (grid resolution 0.234 mm, temporal resolution 1.8E−6 s, 13,605 time steps). The LES allows simulation of the big eddies, while the small eddies are modeled by virtual viscosity. The mesh size must be smaller than the eddy size of the large-scale phenomena and larger than the eddy size of the small-scale phenomena. Simulations were initialized with zero velocity and stopped once the pressure drop between the nostril and oropharynx became stationary with fully developed flow. We considered it stationary once pressure fluctuation was smaller than ± 3%. The outcome of the simulation was the pressure scalar field (Fig. 1b).

### Nasal pressure calculation

To compare the AAR curves with the simulation results (simulated AAR), the pressure drop ${\Delta }p = p1 - p2$ (Fig. 1b) of every single simulation was determined. Since there were 50 ${\Delta }p$ evaluations per subject (25 ${\Delta }p$ evaluations per side, 250 ${\Delta }p$ evaluations for five subjects), this was automatized with a Python script. p1 was determined inside the sphere, p2 in the oropharynx. p1 corresponded to the AAR pressure acquired at the nasal entrance and p2 corresponded to the AAR pressure acquired in the nasopharynx. We observed no severe pressure variation between the nasopharynx and the position of the cuboid (Fig. 1b—color map).

### Data analysis

The digitized data of the congested, decongested and simulated AARs (supplementary material 1) were visualized with Veusz 3.0 (Jeremy Sanders, Garching, Germany). All data were analyzed with the SPSS 24.0 statistical package (SPSS Inc., Chicago, Illinois, USA). The main outcome variables of the analysis were pressure and resistance.

### Pressure

The pressure derived from the simulation was defined as SimPress. Data of the congested and decongested AAR flow rates were reduced to 25 per side by grouping them in steps of 50 ml/s to match with the simulated data (i.e., − 25 to 25, 25 to 75, etc.). Pressure data were aggregated accordingly as the mean values based on grouped flow rates. Of the congested and decongested AARs, the one better matching the simulated AAR was chosen as the clinical AAR. The pressure derived from the clinical AAR was defined as RhinoPress. Data were organized per subject, nasal side and respiration phase (either inspiration or expiration), after being transformed to absolute values. Pressures around 0 Pa with range from − 25 to 25 were removed from the further analysis, since differentiation between inspiration and expiration was essential. To cope with scattering at high-pressure values, pressure data were logarithmically transformed.

The correlation between RhinoPress and SimPress was examined with a univariate analysis of variance. Agreement was investigated with the Bland–Altman method of agreement [16–18] as simultaneously performed by others [10]. We calculated the mean value and difference of the logarithm of RhinoPress and the logarithm of SimPress. The difference between the logarithm of RhinoPress and the logarithm of SimPress is equal to the logarithm of the proportional deviation of RhinoPress from SimPress. Since log_10_(RhinoPress/SimPress) = *x* is equivalent to RhinoPress/SimPress = 10^*x*^, the RhinoPress/SimPress can be easily calculated. We performed an inter-subject examination of agreement per nasal side and respiration phase.

### Resistance

From the data of the congested, decongested and simulated AARs, pressure drops around ${\Delta }p$ = − 150 Pa and ${\Delta }p$ = 150 Pa were chosen. Pressure drops between − 175 Pa and -125 Pa were titled -150 Pa and pressure drops between 125 and 175 Pa were titled 150 Pa. Flow rate data were aggregated accordingly as the mean values based on the ${\Delta }p$ = − 150 Pa and ${\Delta }p$ = 150 Pa. Therefore, 4 ${\Delta }p$ and 4 flow rate values were available for each subject (2 ${\Delta }p$ and 2 flow rate values per nasal side and 2 ${\Delta }p$ and 2 flow rate values per respiration phase). Data were organized per subject, nasal side and respiration phase (either inspiration or expiration), after being transformed to absolute values. Resistance was calculated by dividing the pressure drop (at − 150 Pa and 150 Pa) by the flow rate (ml/s). Only resistance values of inspiration were used for further analysis. The resistance derived from the simulation was defined as SimRes150. The resistance derived from the clinical AAR was defined as RhinoRes150.

The correlation between RhinoRes150 and SimRes150 was examined with a Pearson’s correlation to allow comparison with results of other studies [10]. Correlations were categorized as strong if *r* > 0.8, moderate if 0.6 > *r* > 0.8 and weak if *r* < 0.6. We used a paired t-test for statistical comparison between RhinoRes150 and SimRes150. Similarly, the agreement was investigated with the Bland–Altman method of agreement. We performed an inter-subject examination of agreement per nasal side and respiration phase.

## Results

### Sample

Subjects were 23, 26, 26, 30 and 72 year old. Four subjects were males. In subjects 1, 2, 3 and 4, a right septal deviation was documented. In subject 5, a left septal deviation was documented. In subject 4, the CT was performed 2 h after AAR. In subjects 1, 2, 3 and 5, CT and AAR were performed on different days. In total, 240 SimPress measurements and 196 grouped clinical RhinoPress measurements were available (supplementary material 2). Fewer RhinoPress measurements were available since they were acquired after digitization of original AAR curves that did not always cover the complete flow/pressure spectrum. In total, 10 unilateral SimRes150 measurements and 10 unilateral RhinoRes150 were available during inspiration.

### Pressure

#### Correlation between RhinoPress and SimPress

**Fig. 2:**
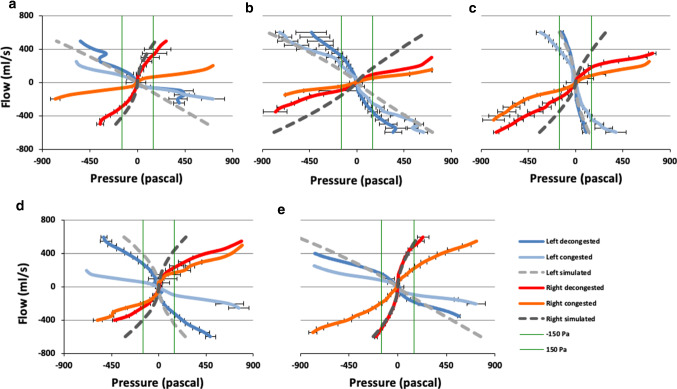
Graphical presentation of the congested, decongested and simulated active anterior rhinomanometry in all subjects. The congested and decongested curves represent the reduced 25 grouped flow rates per side. **a** subject 1; **b** subject 2; **c** subject 3; **d** subject 4; **e** subject 5. *X* axis: pressure in Pascals. *Y* axis: Flow in ml/s. Blue: left decongested; light blue: left congested; red: right decongested; orange: right congested; light gray: left simulated; dark gray: right simulated; green: pressure at − 150 Pa and 150 Pa. Horizontal error bars represent the standard deviation of the aggregated mean values of pressure data

**Fig. 3:**
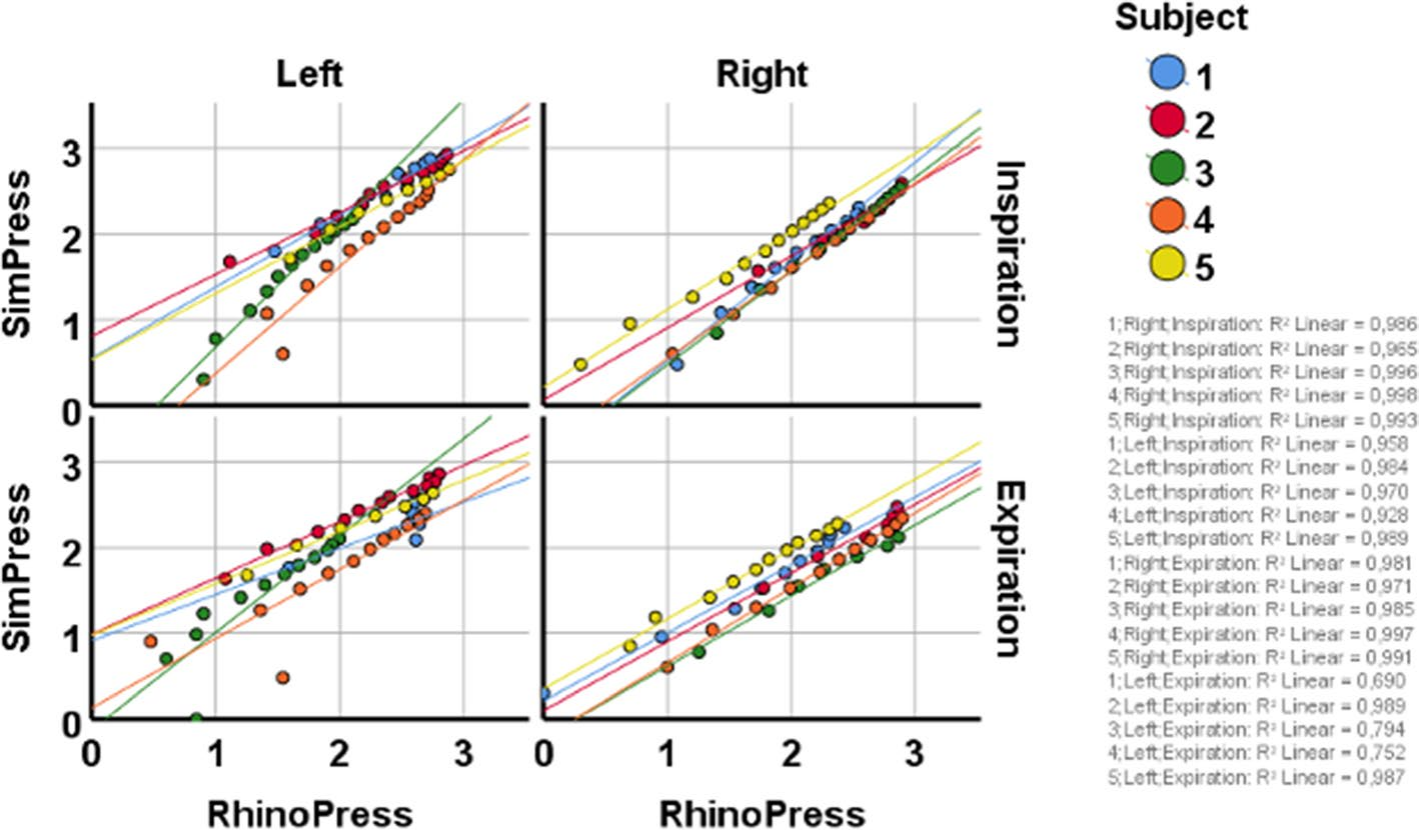
Correlation between pressures derived from simulation (SimPress) and active anterior rhinomanometry (RhinoPress) with a univariate analysis of variance, per subject, nasal side and respiration phase. *X* axis: logarithmic RhinoPress (Pascal). *Y* axis: logarithmic SimPress (Pascal). *R*^2^ linear indicates the square of correlation, e.g., the correlation between SimPress and RhinoPress on the right side of subject 5 (yellow) during inspiration was 0.99 (square root of 0.998)

A visual comparison of the congested, decongested and simulated AAR for all subjects is presented in Fig. 2. The correlation coefficient between RhinoPress and SimPress was *r* = 0.93 (*p* < 0.001), after removing the variations due to subjects, nasal sides and respiration phases. We observed a correlation higher than 0.95 (*p* < 0.001) for 16/20 possible clinical-simulated pressure pairs (for 5 subjects, 2 nasal sides and 2 respiration phases; Fig. 3).

#### Agreement between RhinoPress and SimPress

**Fig. 4:**
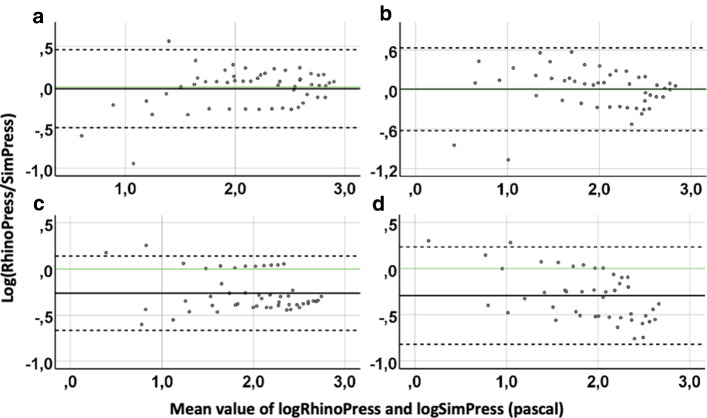
Agreement of pressure derived from active anterior rhinomanometry (RhinoPress) and simulation (SimPress) by Bland–Altman plots in five subjects, per side and respiration phase. *Y* axis: difference of logarithmic RhinoPress and logarithmic SimPress. *X* axis: mean value of logarithmic RhinoPress and logarithmic SimPress in Pascal. Continuous black horizontal line: mean value of differences. Continuous green horizontal line: Best possible agreement (= 0%) between logarithmic RhinoPress and logarithmic SimPress (RhinoPress/SimPress = 1). Upper and lower scattered horizontal line: upper and lower 95% CI of mean value of differences, respectively. **a** Left side during inspiration (54 measurements), **b** left side during expiration (48 measurements), **c** right side during inspiration (49 measurements) and **d** right side during expiration (45 measurements)

On the left nasal side, the mean value ± 95% confidence interval (CI) of the logarithm of the proportional deviation of RhinoPress from SimPress was 2% ± 47% during inspiration and 0% ± 63% during expiration. On the right side, it was 26% ± 40% during inspiration and 29% ± 53% during expiration (Fig. 4; Table 1).

**Table 1:** Proportional deviation of pressure and resistance derived from AAR and the simulation (RhinoPress/SimPress and RhinoRes150/SimRes150, respectively)

Side	Respiration phase	Pressure			Resistance		
		*N*	Logarithmic proportional deviation (%)^a^	RhinoPress/SimPress^a^	*N*	Logarithmic proportional deviation (%)^b^	RhinoRes150/SimRes150^b^
Left	Inspiration	54	2 ± 47	1.05 ± 2.95	–	–	–
	Expiration	48	0 ± 63	1.00 ± 4.26	–	–	–
Right	Inspiration	49	26 ± 40	1.82 ± 2.51	–	–	–
	Expiration	45	29 ± 53	1.95 ± 3.38	–	–	–
Unilateral	Inspiration	–	–	–	10	3 ± 34	1.07 ± 2.17

^a^Mean ± 95% confidence intervals from the Bland–Altman agreement of measurements, e.g., on the left side of all subjects during inspiration, the mean logarithm of the proportional deviation of RhinoPress from SimPress was 2% ± 47% for 54 measurements. This implied that the RhinoPress was 1.05 ± 2.95 times greater than the SimPress

^b^Mean ± 95% confidence intervals from Bland–Altman agreement of measurements, e.g., by all subjects during inspiration, the mean logarithm of the proportional deviation of RhinoRes150 from SimRes150 was 3% ± 34% for 10 measurements. This implied that the RhinoRes150 was 1.07 ± 2.17 times greater than the SimRes150

After applying the equations log_10_(RhinoPress/SimPress) = *x* and RhinoPress/SimPress = 10^*x*^, RhinoPress was found to be 1.05 ± 2.95 times greater than SimPress on the left side during inspiration and 1.00 ± 4.26 times greater during expiration. On the right side, RhinoPress was 1.82 ± 2.51 times greater than SimPress during inspiration and 1.95 ± 3.38 times during expiration (Fig. 4; Table 1).

### Resistance

#### Correlation between RhinoRes150 and SimRes150 during inspiration

**Fig. 5:**
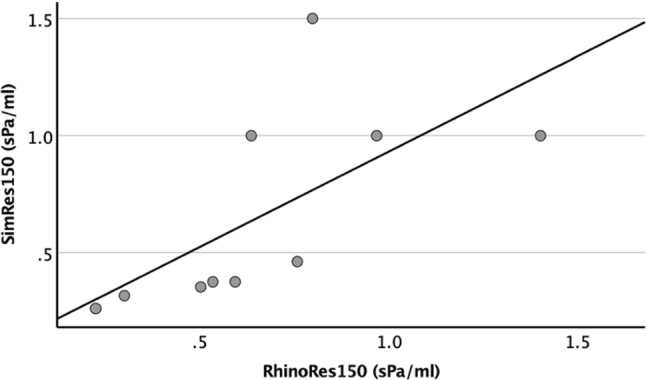
Correlation between resistances by 150 Pa pressure drop during inspiration derived from simulation (SimRes150) and active anterior rhinomanometry (RhinoRes150) with Pearson’s correlation. *Y* axis: SimRes150 (sPa/ml). *X* axis: RhinoRes150 (sPa/ml). The diagonal line is the best fit line at total (*r* = 0.65)

A visual comparison of the congested, decongested and simulated AAR for all subjects is presented in Fig. 2 (green vertical line). A moderate correlation was found between unilateral RhinoRes150 and unilateral SimRes150 during inspiration (*r* = 0.65, *p* = 0.041; Fig. 5). The mean value ± standard deviation (SD) of the unilateral RhinoRes150 (0.67 ± 0.34 sPa/ml) was similar to that of the unilateral SimRes150 (0.66 ± 0.43 sPa/ml; *p* > 0.2).

#### Agreement between RhinoRes150 and SimRes150 during inspiration

**Fig. 6:**
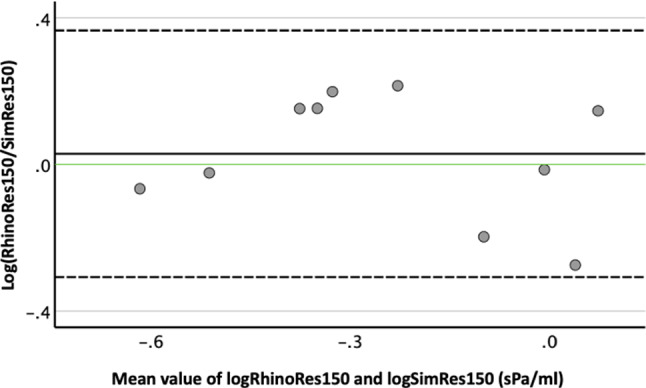
Agreement of unilateral resistances (10 in total) by 150 Pa pressure drop during inspiration derived from active anterior rhinomanometry (RhinoRes150) and simulation (SimRes150) by Bland–Altman plot in five subjects. *Y* axis: difference of logarithmic RhinoRes150 and logarithmic SimRes150. *X* axis: mean value of logarithmic RhinoRes150 and logarithmic SimRes150 in sPa/ml. Continuous black horizontal line: mean value of differences. Continuous green horizontal line: Best possible agreement (= 0%) between logarithmic RhinoPress and logarithmic SimPress (RhinoPress/SimPress = 1). Upper and lower scattered horizontal line: upper and lower 95% CI of mean value of differences, respectively

The mean value ± CI of the logarithm of the proportional deviation of unilateral RhinoRes150 from unilateral SimRes150 was 3% ± 34% during inspiration. After applying the equations log_10_(RhinoRes150/SimRes150) = *x* and RhinoRes150/SimRes150 = 10^*x*^, RhinoRes150 was found to be 1.07 ± 2.17 times greater than SimRes150 during inspiration (Fig. 6; Table 1).

## Discussion

In this Pilot study, we investigated whether the clinically performed AAR could correlate with a simulated AAR generated from CT-based CFD. For this purpose, we compared the pressure values derived from clinical AAR and simulated AAR. Our results revealed an excellent correlation between pressures derived from both methods (*r* = 0.93; *p* < 0.001), after removing the variations due to subjects, nasal sides and respiration phases. However, this was a trivial, even misleading finding, as the flow rate necessarily increases when the pressure drop increases. In addition, the high number of observations increased the observed correlation. To cope with this issue, we compared the more commonly clinically used resistance by 150 Pa pressure drop during inspiration, derived from clinical and simulated AAR. Our results revealed a moderate correlation between resistances derived from both methods (*r* = 0.65, *p* = 0.041; Fig. 5).

Since correlation does not necessarily indicate agreement, we further investigated this issue by the Bland–Altman method in line with similar studies [10]. This statistical method is used to assess agreement between two methods of measurements with repeated data. To precisely describe how smaller or greater pressure and resistance values of AAR were in comparison with pressure and resistance values of the simulation, we logarithmically transformed the pressure and resistance values. The *Y*-Axis of the Bland–Altman plot was the difference between two methods of measurements. The difference of logarithmic RhinoPress and logarithmic SimPress is equal to the proportional deviation of logarithmic RhinoPress and logarithmic SimPress. This led to easy calculation of the proportional deviation of RhinoPress and SimPress. Similarly, the same approach was followed for the RhinoRes150 and SimRes150.

The proportional deviation of pressure values was computed separately for each nasal side and respiration phase. A low value for the logarithm of the proportional deviation would indicate a high degree of agreement. Our results revealed a logarithm of the proportional deviation of 1–29% (Table 1). This implied that the RhinoPress was 1–2 times greater than SimPress (Table 1). This variation was more crucial for higher ${\Delta }p$. This suggested an inverse proportionality (“the lower the ${\Delta }p$, the higher the agreement”). Similarly, the documented value for the logarithm of the proportional deviation of RhinoRes150 from SimRes150 was 3%, which implied that the RhinoRes150 was similar to the SimRes150.

Substantial side predominance was noted. On the left side, RhinoPress was similar to SimPress. On the right side, RhinoPress was two times greater than SimPress. Septal deviation to the right was present in 4/5 subjects. These results could indicate a better agreement on the less obstructed and a worse agreement on the more obstructed nasal side. Indeed, the agreement between RhinoPress and SimPress for subject 5 (left septal deviation) was slightly better on the right side (5.0%) than in the left side (5.3%; data not shown).

Our results imply that the CT-based CFD with the LB code could roughly predict the pressure and resistance calculated by the AAR, at least on the less obstructed nasal side. Moreover, the resistances during inspiration by a 150 Pa pressure drop derived from both methods were comparable (*p* > 0.2). Also, agreement between RhinoRes150 and SimRes150 was high. However, error rates of up to 100% were encountered on the more obstructed nasal side. Furthermore, even in the less obstructed nasal side, error rates of up to 400% might occur, e.g., the RhinoPress was 1.0 ± 4.26 times greater than SimPress on the left nasal side during expiration. These results imply that the pressure and resistance values derived from AAR and CT-based CFD are similar, yet not same.

The first problem we encountered was the mucosal status. We were obliged to choose either the congested or decongested AAR as clinical AAR. We based this decision on the agreement between either the congested or the decongested AAR and the simulated AAR. This agreement depended on the nasal mucosa status. The simulated AAR was generated from a CT scan. At the time of the CT, the nasal mucosa was in either a congested or a noncongested state. This depended on the nasal cycle, resulting in one congested and one noncongested state of the nose. Therefore, we noted a high agreement between the simulated AAR and either the congested or the decongested AAR, but not both. Indeed, visual inspection of the congested, decongested and simulated AARs (Fig. 2) supported this observation.

In all subjects, AAR was performed according to international standards [1], and the same imaging protocol (DVT) was chosen. DVT provided approximately 400 DICOM files in total for the axial, coronal and sagittal planes, substantially useful for application in CFD. With the lattice Boltzmann package Sailfish CFD, the segmented 3D data structure in DICOM file format can be used for simulations without any preprocessing. Sailfish CFD is numerically stable with the complex shapes of the nasal airway passage in the airflow range of AAR.

The digitization of the AAR pdf data resulted in pixel-based pictures with a pixel density of 200 dots per inch (dpi). For printing, 300 dpi is standard; 150 dpi is considered acceptable to separate raw AAR data curves [19]. For data extraction, RGB color thresholds with ± 10 range were chosen. The color was specified by visual selection with the graphical user interface pipette. The colors of the AAR curves were unique in the original pdf file; a larger threshold range provided no additional benefits. The extracted AAR analysis depended on the discretization window applied to the original pdf file; a discretization window of 5 × 5 pixels resulted in an extracted AAR resolution of more than 1500 *x*–*y* points. For this, the computational time was less than a second. A larger discretization window with less computational time—but also with lower AAR resolution—was not considered. Since the extracted AAR resolution was considered acceptable, a smaller discretization window with an even higher extracted AAR resolution was not needed.

The nasal air space extraction was programmed in Python and performed based on thresholding at -460 HU [13]. The simple thresholding segmentation led to occasional artificial connections between the three nasal meatus, artificial septal perforations or bad connectivity to paranasal sinuses in anatomical sites with very thin tissues. Here, a slight effect at the results cannot be ruled out. This might explain the worse agreement between AAR and CT-based CFD on the more obstructed nasal side, in which the borders between structures are not as easily outlined as on the less obstructed nasal side. The simulation of the airflow at the nasal tip was critical [20]. For this reason, a sphere was positioned at the nasal tip. The sphere extended the simulation area without ‘hurting’ the anatomical integrity of the anterior nose, which is crucial for the airflow. Therefore, the airflow at the nasal tip reflected real-life conditions. We set the sphere diameter to 70 mm [21]; a larger diameter would merely increase the computational time without any additional advantages, and a smaller diameter might influence the integrity of the anterior nasal structures. The cuboid at the outlet was considered the appropriate geometrical element to define flow rate $\dot{V}$ by a “Dirichlet” velocity boundary condition (constant velocity at the outlet boundary condition). The Python script allowed automatic segmentation without user interaction for all CT datasets. One single manual correction was performed in order to reflect AAR conditions. Specifically, one nostril was locked manually during simulation.

The accuracy of the LB fluid flow simulations depends on the fluid properties and the resolution of the spatial discretization. Air was simulated with a constant temperature of 20 °C, which was specified by a constant density and viscosity. During inspiration, the air temperature increases, which results in an increase in viscosity and a reduction in density. This change is not reflected within the LB simulation [22]. The resolution of the spatial discretization was considered sufficient and mesh independent [11]. However, the resolution was not sufficient to resolve sub-millimetric turbulent flow phenomena, especially when the air-space cross section was smaller than 3–5 cells (0.702–1.17 mm). In our study, we focused on nasal airflow in general. We did not perform a turbulent spectrum analysis. We performed LDA measurements to determine the accuracy of the velocity vector field and a mesh convergence study to guarantee that the pressure between sphere and oropharynx was independent of the grid size. Within the simulations, pressure extraction was performed in the sphere and the cuboid in the oropharynx (Fig. 1b). Significant pressure changes were not observed in either the sphere or the cuboid. This reassures the accuracy of the pressure extraction. Moreover, the computational time of the simulation using Sailfish CFD on a NVIDIA GTX 980 Ti was less than six minutes. Furthermore, the pressure drop is an extremely robust parameter, tolerant at coarse meshes. Despite the nose complex geometry, the nasal airflow is a simple fluid dynamical problem.

In contrast to Cherobin and coauthors [10], we examined the pressure scalar field. The investigation was performed with the LB code. Here, the air segmented CT datasets are used without any further meshing process, which simplifies the workflow [14]. The LB code is simple and fast when run on a GPU [14]. The LES Smagorinsky subgrid model used is more accurate than k-omega [23]. The disadvantages of LB include greater memory usage and numerical stability only in a defined velocity range [14]. Furthermore, Cherobin and coauthors compared 50 left and right resistance measurements and 50 left and right conductance measurements of 25 patients, derived from CFD and AAR, also by the Bland–Altman method of agreement. The authors reported a weak to moderate correlation between CFD and AAR. The comparison was performed only at ${\Delta }p$ = 75 Pa [10]. In our study, we compared the complete clinical AAR curve with the complete simulated AAR curve. Here, we analyzed grouped pairs of flow and pressure measurements that covered the complete spectrum of flow (− 625 to 625 ml/s) and pressure (Fig. 2; supplementary material 1 in raw form), thus presenting a more representative comparison of both methods. Obviously, ${\Delta }p$ = 150 Pa or near it remains the most useful clinical measurements [24]. Therefore, we also examined the resistance by ${\Delta }p$ = 150 Pa during inspiration. Here, we noted a moderate correlation between CFD and AAR. Resistance values did not differ significantly between both methods. However, this might be related to the small sample size, since we noted a tendency for lower resistance values in CFD than in AAR.

Limitations of our pilot study included the small sample size and the effect of the nasal cycle. Initially, we intended to investigate the correlation and agreement of CT-CFD and AAR by a new approach. Most steps of the processing pipeline were automated and did not require much computational power. Usually, pilot studies investigate small samples for feasibility. The first results were the graphical presentation of simulation data and AAR data (similar to Fig. 2) that indicated a promising visual agreement. For statistical investigation, AAR data were digitized, aggregated, redistributed and analyzed. This was an elaborate process, since we compared paired values of flow and pressure of the complete flow/pressure spectrum and not only around ${\Delta }p$ = 150 Pa. Furthermore, the limited funding for this preliminary investigation did not allow increasing the study sample size.

In conclusion, despite the advantages of the LB simulation, our results were close to the results described by other studies. The pressure and resistance values derived from CT-based CFD and AAR were similar, yet not same. The presence of a sphere at the tip of the nose ensured the anatomical integrity of the anterior nose. The LB simulation was applicable for the complicated geometry of the nose and required low computational time. Most importantly, the LB simulation was validated with LDA to assure the validity of the simulation approach. Improvements may include a larger sample size and the decongestion of the nasal mucosa.

## Supplementary Information

Below is the link to the electronic supplementary material.

Supplementary file1 (XLSX 1051 KB)

Supplementary file2 (SAV 23 KB)

## Declarations

### Availability of data and material

All data generated or analyzed during this study are included in this published article (and its supplementary information files).
